# Hospital discharge of elderly patients to primary health care, with and without an intermediate care hospital – a qualitative study of health professionals' experiences

**DOI:** 10.5334/ijic.1156

**Published:** 2014-04-30

**Authors:** Unni Dahl, Aslak Steinsbekk, Svanhild Jenssen, Roar Johnsen

**Affiliations:** Department of Public Health and General Practice, Norwegian University of Science and Technology, 7491 Trondheim, Norway; Central Norway Health Authority, 7500 Stjørdal, Norway; Department of Public Health and General Practice, Norwegian University of Science and Technology, 7491 Trondheim, Norway; Central Norway Health Authority, 7500 Stjørdal, Norway; Department of Public Health and General Practice, Norwegian University of Science and Technology, 7491 Trondheim, Norway

**Keywords:** intermediate care hospital, hospital discharge, elderly, integrated care, coordination, professional communication

## Abstract

**Introduction:**

Intermediate care is an organisational approach to improve the coordination of health care services between health care levels. In Central Norway an intermediate care hospital was established in a municipality to improve discharge from a general hospital to primary health care. The aim of this study was to investigate how health professionals experienced hospital discharge of elderly patients to primary health care with and without an intermediate care hospital.

**Methods:**

A qualitative study with data collected through semi-structured focus groups and individual interviews.

**Results:**

Discharge via the intermediate care hospital was contrasted favourably compared to discharge directly from hospital to primary health care. Although increased capacity to receive patients from hospital and prepare them for discharge to primary health care was viewed as a benefit, professionals still requested better communication with the preceding care level concerning further treatment and care for the elderly patients.

**Conclusions:**

The intermediate care hospital reduced the coordination challenges during discharge of elderly patients from hospital to primary health care. Nevertheless, the intermediate care was experienced more like an extension of hospital than an included part of primary health care and did not meet the need for communication across care levels.

## Introduction

In delivering safe and effective health care services, lack of coordinated services during transition between hospital and home is a major concern in the Norwegian health policy [1] as in other Western countries [2–5]. Large European projects are set up to optimise the continuity of health care at the primary care – hospital interface [6]. Currently, discharge from hospital is considered as a critical phase with increased risk of adverse events and readmission, especially for elderly with multiple health problems [7–9]. The increasing number of older patients, shorter hospital stays and demands for services closer to home emphasise the need for collaboration between hospital and primary health care [10–12]. However, several studies have identified insufficient communication and collaboration failure between organisations and health care personnel during hospital discharge [13–16].

Integrated care strategies can be defined as techniques and organisational models designed to create connectivity, alignment and collaboration within and between the cure and the care sectors [17]. Different concepts such as transitional care [4], care pathways and integrated care pathways [18] are also used for the approach to strengthen coordination of health care services when patents transfer between different locations, providers or levels of care. Integrated discharge pathways for elderly patients can be achieved in various ways: Discharge arrangements range from hospital-based teams [19], care pathways across hospital and primary health care [20, 21], to interventions within primary health care to improve discharge support [22]. A combination of pre- and post-discharge interventions seems to be essential [23], as this recently has been pointed out among features of successful discharge interventions targeted at elderly patients [24]. Discharge coordinator, multidisciplinary approach, education, medical reconciliation and comprehensive transitional care programmes are other identified features of success [24].

Intermediate care services have been an approach to bridge the gap between hospital and primary health care for more than a decade [25, 26]. They comprise a heterogeneous group of services such as nurse-led units, hospital-at-home, nursing home-based rehabilitation, rapid response teams and community hospitals [27, 28]. The main objective of intermediate care, as in integrated care, is to improve continuity of care for elderly patients by enhancing coordination and collaboration between care systems and providers [27, 29]. The key functions are to prevent unnecessary hospital admission, support timely discharge from hospital and maximise independent living [1, 25].

It has been shown that intermediate care can contribute to avoid hospital admission, support early discharge and enable patients to regain abilities in daily living [30–34]. Nevertheless, intermediate care faces challenges [27, 35]. Regen et al. interviewed managers and practitioners working within or relating to intermediate care services in England [36]. They identified collaboration between organisations and professionals [37, 38] and integration with the ‘mainstream’ services [39] as challenges to developing intermediate care. Mur-Veeman and Govers [40] found that although stakeholders see the importance of integrated care, factors like lack of willingness and ability along with their perceptions, routines, principles and beliefs hindered the necessary cooperation.

Due to vague definitions [41] and challenges integrating intermediate care with the mainstream services, more knowledge of the intermediate care models in various settings is needed. Exploring professionals’ experiences with an intermediate care model compared to experiences with usual care would extend the understanding of strengths and limitations of intermediate care services.

This is a study of an intermediate care hospital established in Central Norway to improve discharge from a general hospital to primary health care. The aim of the study was to investigate how professionals across health care levels experience the discharge of elderly patients, who are in need of continued care, from a general hospital via an intermediate care hospital compared to a direct discharge to primary health care in a municipality without intermediate care.

## Methods

The Regional Committee for Medical Research Ethics approved the study (2009/1697a). The data collection took place from September 2010 to October 2011. The informants participated based on informed consent.

### Design

This was a qualitative study using semi-structured focus groups and individual interviews, observation and document review. The design was chosen as qualitative methods are recommended to explore experiences and attitudes in depth [42, 43]. The combination of methods was used to give a comprehensive picture of the discharge situation: Focus groups were conducted to explore the professionals' discussions regarding views and experiences. Because some informants were unable to attend the focus groups, individual interviews were performed. Observations provide data from real-life settings and were carried out mainly to get a better understanding of the informants’ situation and to clarify facts and discussions in the interview transcripts. The purpose of the document review was to learn about the background of and arrangements for the intermediate care hospital. A checklist for reporting qualitative interviews was used as a guide [44].

Furthermore, the design involved interviewing informants about two different situations: discharge from the hospital to primary care with and without an intermediate care hospital. Comparing the experiences from two different municipalities did this.

### Setting

Norwegian health and social care combines financing and provision of services mainly within the public sector and is organised in primary and specialist health care services [45]. Primary health care services are the responsibility of the municipalities and include general practitioners, home care services and nursing homes. The hospitals (specialist services) are state-owned and are operated by four regional health authorities.

In 2007, a municipality of 22,000 inhabitants in Central Norway established an intermediate care service in collaboration with the general hospital and the regional health authorities [46, 47] called an intermediate care hospital. It is an in-patient ward with 12 beds colocated with primary health care services and is situated in a municipality other than the hospital which is approximately 50 km away. The services are targeted at patients discharged from the general hospital who require resources above the level delivered by primary health care. The goals are to develop an integrated care pathway for elderly and chronically ill patients, and an arena for professional collaboration with exchange of information and knowledge between the general hospital and primary health care [46].

The hospital physician decides when a patient is ready for discharge to intermediate care and usually a hospital nurse requests a place by phone. The patients are discharged to the intermediate care hospital on the same or next day and at an earlier stage compared to discharge directly to primary health care services. The agreement is that intermediate care shall follow up patients with complex illnesses and comprehensive care needs, and those who need a couple of extra days of institutional treatment after diagnostics and completion of the initial treatment in the general hospital by, e.g. providing intravenous treatment. To prevent prolonged stay at the general hospital, the intermediate care hospital had gradually adjusted the criteria for admittance. The mean length of stay at the intermediate care hospital was approximately 10 days.

The intermediate care hospital is staffed with mainly nurses of whom many are specialised and have previous experience in hospital work. In addition, there are occupational therapists and physiotherapists, and a general practitioner is present during weekdays. The intermediate care staff emphasises follow-up treatment, activities of daily living, motivation and promotion of safety to enable as many patients as possible to return home. Representatives from the intermediate care management and the admission unit for primary health care services have daily meetings. Also regular multidisciplinary meetings between the intermediate care staff and primary health care are held to optimise the discharge process. The intermediate care hospital uses the same electronic health record as the primary health care in the municipality. The general practitioner and the intermediate care staff can consult the general hospital as needed by phone or by videoconference, and there is collaboration with the general hospital about procedures and training of the intermediate care staff. Some of the employees at the intermediate care hospital also have reading access to the general hospital's electronic health record.

The comparative municipality of 14,500 inhabitants, which does not have an intermediate care hospital, is also located in the catchment area of the same hospital. The usual discharge process to municipalities without intermediate care starts when a hospital physician decides that the patient is ready to be discharged. If patients are assessed to be in need of post-hospital care, the hospital nurses notify one or more contact persons in the patient's municipality by a written application and by phone to initiate home care services or a nursing home. Once primary health care has decided the patient's level of care and the services that are available, the patient is discharged. However, there is often a discussion between the hospital and the primary health care about the appropriate time for discharge.

### Participants

A purposeful sampling of personnel was done to ensure coverage in health professional and organisational representation [42]. A contact person at each study site recruited informants who were familiar with the discharge process. Four focus group interviews were conducted, in the general hospital, the intermediate care hospital, and the primary health care services with and without an intermediate care hospital. Each group consisted of five–nine participants. Additionally, three individual interviews were conducted.

### Data collection

The first author, UD, conducted the interviews, the onsite observations and the document review. Author RJ was present as a comoderator at the first focus group interview. All focus group interviews, which lasted from 60 to 90 minutes, were audio-recorded and transcribed verbatim. One of three individual interviews was audio-recorded and transcribed. Data from the other two were recorded by writing notes as they took place during opportunistic situations and thus no recorder was present: The first was conducted by telephone and the second during observation.

To ensure that the informants discussed the same themes, a semi-structured interview guide was used. The guide was developed before data collection started, and was used to ensure that all topics were covered in the interviews. The interviewer introduced the themes from the interview guide if the informants did not spontaneously discuss them. The informants were also free to talk about aspects beyond the guide. The core research question was ‘What are your experiences with discharge of patients from the general hospital?’ The focus group in the general hospital and the municipality with an intermediate care hospital was asked about experiences of discharge with and without an intermediate care hospital. Another important topic in the interviews was how the informants interacted with professionals in other locations. The informants were also asked to describe the course of events during discharge.

The observations were conducted after the interviews. They were performed at two hospital departments during day shift and included conversations with nurses, physicians and secretaries about experiences and routines. A similar observation was performed at the intermediate care hospital. Field notes were written continuously during the observations. The interview guide was used as a guide in the observations. In the same period, some informants from primary health care (participants in previously conducted focus groups) were asked more specific questions. This was done to verify that data in their interview transcripts were properly understood.

The document review consisted mainly of reading written case documents regarding the establishment of the intermediate care hospital, especially the setup of the organisation and objectives. Additionally, discharge criteria to intermediate care and information forms for discharge were studied.

The observations confirmed that saturation was reached as no new views, experiences or facts emerged.

### Analysis

The data were analysed by systematic text condensation as described by Malterud [48]. Systematic text condensation is a stepwise analysis suitable for data from interviews, observations and written texts, inspired by phenomenology and developed from traditions shared by most of the methods for analysis of qualitative data [49]. First, all authors read the focus group interviews independently to get an overall impression and identify main themes that addressed the experiences with and without an intermediate care hospital at discharge. The authors then met to discuss the main themes. Thereafter, first author UD read and ensured that the themes from the individual interviews, supplemented by field notes and documents, were included in the analysis, and she identified units of meaning and subthemes within the agreed main themes. The main themes and subthemes were discussed and revised in two subsequent meetings. Next the content of the subthemes was condensed and thereafter, the content of each subtheme was summarised to generalise descriptions and concepts of the health professionals’ experiences of discharge. Rereading the interviews tested the relationship between the themes. The results were presented to some participants in the focus groups to check for possible misinterpretations. To select citations to illustrate the themes, the citations from the same themes were compared and those that were the most illustrative for each of the themes were selected.

## Results

A total of 27 people were interviewed (Table 1). The informants were professionals and managers from the general hospital, the intermediate care hospital and primary health care services with and without an intermediate care hospital.

### Views and experiences at discharge of elderly patients in need of continued care

**Figure 1.**
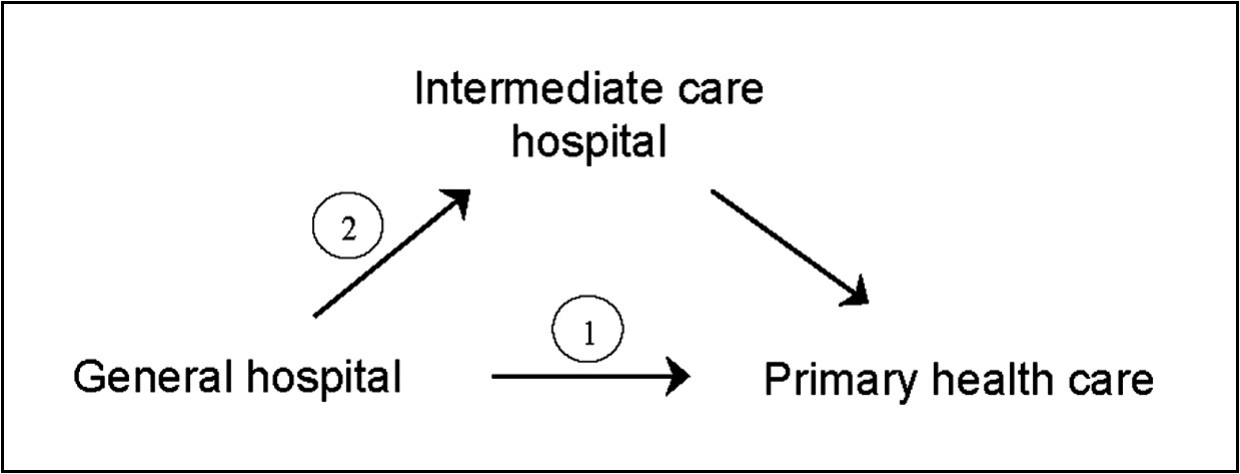
Discharge with and without the intermediate care hospital. 1) Discharge from the general hospital to primary health care without an intermediate care hospital. 2) Discharge from the general hospital via an intermediate care hospital to primary health care.

The views and experiences of discharge with and without an intermediate care hospital were clarified by comparing the two care pathways from the general hospital to primary health care (Figure 1). The experiences at discharge without an intermediate care hospital are presented from the views of the general hospital and primary health care. These findings were compared to the experiences with an intermediate care hospital in the pathway of discharge, and first elaborated in the views of the general hospital, then primary health care and finally the intermediate care hospital.

### Experiences without an intermediate care hospital

These findings are based on information collected in the general hospital and the comparative municipality without an intermediate care hospital (Figure 1). However, the circumstances are similar to the situation before intermediate care was established in the municipality with the intermediate care hospital.

#### Views of the general hospital

During discharge without an intermediate care hospital, the informants from the general hospital requested sufficient capacity in primary health care to handle patients ready for discharge as soon as the diagnostics and hospital treatment were completed. However, one informant said

When we realise that there is obviously too low a level of care in primary health care, we keep the patient. For that reason. (Hospital physician)

Among physicians and nurses in the hospital, there was a mutual understanding that primary health care services often lacked the necessary structure and resources to receive elderly patients with comprehensive care needs. Some nurses said that it seemed like primary health care did not prepare for the return of patients during hospitalisation (Box 1).

The informants were also familiar with unnecessary acute admissions of elderly due to general reduced health, social situations and lack of adequate resources in primary health care. To obtain adequate care for patients, home care or the patient's caregivers might even ask the hospital, on admission, to request increased local resources, for instance, a nursing home.

Regarding communication at discharge, the hospital physicians considered the discharge summary to the general practitioners to be sufficient, while some nurses felt a need for an extended communication with primary health care to arrange for post-hospital care. This was due to an increasing number of patients in need of assistance to care for themselves. The hospital nurses also said it was important to be available on phone after discharge of the patients in order to clarify information about procedures and medications to primary health care workers.

#### Views of primary health care services

The informants from primary health care without an intermediate care hospital considered it essential to get information in advance of discharge to enable them to assess the patient's functional status, arrange for resources and the best place of care. This was related to the home care nurses experiences of incomplete information from the general hospital and lack of daily living aids when the patient arrived home.

We get that discharge summary with medical treatment. […] In nearly 80% of the cases we have to call [the hospital] to clear it up: Are they supposed to have this medication or not? It's not listed. Is it ceased? (Home care nurse)

According to the home care nurses, also some elderly patients would benefit from additional time in the hospital.

I wonder – the patient is barely out of the hospital bed when he comes home. Many are too weak to stay at home. Some patients are in such poor condition when they get home, that they are returned to the hospital through the emergency department. (Home care nurse)

The insufficient preparations caused a rush to get resources and medication in place and impeded the care of the patient. Thus, the home care nurses felt they had to check and request supplementary information each time they were contacted by the hospital prior to discharge – ‘like a watchdog’ one nurse remarked. All informants from primary health care expressed a wish for communication with the hospital before discharge, better planning and collaboration with routines and procedures to ensure proper preparations for support locally.

### Experiences with an intermediate care hospital

The implementation of the intermediate care hospital was said to change both the capacity to handle patients ready for discharge and the preparations for discharge to primary health care services (Figure 1).

#### Views of the general hospital

The informants from the general hospital perceived the intermediate care hospital as an extension of a hospital department.

I think it's before primary health care […] More hospital than the primary care and a little less hospital than specialist care. I consider it as a simplified hospital department. (Hospital physician)

The discharge of patients was uncomplicated and resembled a transfer between departments. A prominent benefit seen from the hospital perspective was that the intermediate care hospital had a capacity that provided early and timely discharge (Box 1). Discharge to the intermediate care hospital, by contrast to earlier practice of discharge to long-term care or nursing home, was viewed as a much more available alternative.

Many patients are sent to the intermediate care hospital and the intermediate care hospital takes over the collaboration with primary health care. This greatly reduces work in the department. The department is collaborating well with the intermediate care hospital, the intermediate care manager participates in hospital meetings and we understand each other well. (Hospital nurse)

#### Views of primary health care services

The informants from primary health care perceived the intermediate care hospital as a buffer that provided preparations for discharge of the patients. The intermediate care hospital had clearly improved the transition process compared to direct discharge from the general hospital.

Home care nurse: “After intermediate care hospital was established, it is perhaps the planning of daily living aids before they return home that has been improved the most. […] I assume the hospital is not ordering much equipment.”

Occupational therapist: “No, they don't.”

Primary health care highlighted that intermediate care shielded them from the fast discharge from the general hospital of elderly patients with comprehensive care needs. They were also satisfied with the patients’ improved condition after a stay at the intermediate care hospital. A home care nurse emphasised how the intermediate care staff motivated and pushed the patients to achieve independence in such a way that some patients could return home without assistance from home care.

Informants from the admission unit said that sharing of the electronic health record gave primary health care direct access to information, and thus made it possible to gain knowledge about their patients before discharge. Nevertheless, when there were no vacancies at the intermediate care hospital and the general hospital requested a bed, the home care nurses and their colleagues said that they had to receive a patient within the same day as they were informed about the discharge from intermediate care. This forced them to rapidly change their schedules to give priority to the discharged patient. Thus, there were discharges from the intermediate care hospital that resembled discharges directly from the general hospital.

It was expressed that the communication regarding discharge of patients to primary health care had improved greatly with the implementation of the intermediate care hospital.

Admission unit: “It has improved a good deal, but it is still a way to go. They live their own lives in a way there after all.”

Interviewer: “Why has it improved?”

Admission unit and home care nurse: “The dialogue.”

Despite this improved situation, the informants from primary health care still experienced a lack of mutual understanding of their role and tasks; hence, they felt that there still was room for an improved professional communication with the intermediate care staff. The home care nurses who said that they had repeatedly requested to participate in the multidisciplinary meetings at the intermediate care hospital exemplified this. They considered their knowledge of patients with comprehensive care needs as important in the planning and discharge preparations.

#### Views of the intermediate care hospital

The informants from the intermediate care hospital perceived their role as an efficient discharge unit preparing patients and arranging for them to live home (Box 1). They were satisfied with the relationship to the general hospital and agreed on the importance of receiving as many patients as possible. However, occasionally, the intermediate care staff experienced that patients arrived without having received information of what to expect during the stay. Additionally, even if reading access to the electronic health record in the general hospital was an important information source, the informants said that they quite often had to ask for supplementary information from the general hospital, usually by phone.

The attention to support early and timely discharge caused an increased pressure in intermediate care to receive patients from the general hospital. This meant that the intermediate care hospital had to focus on discharging patients to primary health care. The management at the intermediate care hospital said that they balanced between maximising the patients’ functions and the unit's capacity when deciding on the patient's discharge.

The primary health care prefers the patients to stay as long as possible. So then we have to push the primary health care services and say: This patient is actually ready for discharge. (Intermediate care manager)

Nevertheless, the informants at the intermediate care hospital thought the coordination with primary health care regarding discharge was working reasonably well. This was especially due to sharing information by the electronic health record and the daily meetings between the intermediate care staff and the primary health care unit responsible for admission and allocating services.

## Discussion

The discharge process of elderly patients via the intermediate care hospital was clearly improved compared to discharge directly from the general hospital to primary health care. The informants at the general hospital experienced the intermediate care hospital as an extension of a hospital department; the informants from primary health care viewed it as a buffer; the informants from intermediate care perceived their role as an efficient discharge unit in close collaboration with the general hospital.

### Strengths and limitations

The main strength of this study is that it included informants who viewed the intermediate care hospital from different perspectives. In addition, interviews were supplemented by observations, thus giving a comprehensive picture of the discharge situation. A weakness was that at each study site, a contact person recruited informants for the focus groups. This could have included informants who had a special agenda. However, the observations carried out showed that those taking part in the interviews had views similar to other staff members. Another weakness was that neither general practitioners nor patients were interviewed. They would have contributed with further experiences about the discharge situation.

The data collection for this study was completed two months before the implementation of the Norwegian health reform (The Coordination Reform) 1 January 2012 [1]. One of the main incentives was that for patients considered ready for discharge by the hospital, the municipalities must pay NOK 4000 (approximately, EUR 500) for each subsequent hospital day. This is the likely reason for decreased hospitalisation time for patients ready for discharge in 2012 [50]. However, the knowledge of services such as intermediate care hospitals is essential to achieve sufficient collaboration and coordinated services during the discharge process.

The reform is a long-term action where the effects will take place when the municipalities have the measures that reduce the need for hospital use. It is too early to draw firm conclusions, and the effects of the reform need to be studied further.

### Experiences at discharge without intermediate care

The analysis of discharge without an intermediate care hospital revealed challenges in the communication and collaboration between the general hospital and primary health care services in line with current knowledge. A prominent finding, described in previous research, was the delayed discharge from the hospital of persons with comprehensive care needs [51]. Furthermore, a different perspective [21] and thus a different understanding of the term ‘ready to be discharged’ appeared between the hospital and the primary health care [13, 52]. It is known from previous studies that some discharge processes occur before support mechanisms can respond in a timely manner [11, 53]. Others have also found that professionals in each setting tended to operate without sufficient knowledge of the services provided in previous settings [7, 14, 16]. A recent article stated that the discharge information from an hospital is out of phase with the primary health care tasks it is supposed to support [54]. Hence, several of the findings in this study regarding discharge without an intermediate care hospital confirm the view that patients’ needs for coordinated services are not being sufficiently met [1, 15].

### Experiences of the role of intermediate care hospitals

There was a consensus that intermediate care hospital contributed to bridge the gap between the general hospital and primary health care services. It provided the capacity to handle patients ready for discharge from the general hospital and introduced simplified discharge routines. It also prepared for admission to primary health care, particularly by improving the patients’ condition, the provision of daily living aids and the required information. The intermediate care hospital's role as a discharge unit includes several features for successful discharge [24] and is in line with Morris [11] who stated that rapidly moving patients through the emergency system towards discharge is an approach that may benefit younger people at the expense of effective planning and comprehensive treatment for the frail and elderly.

In the present study, there was no indication of what Plochg et al. found [37] regarding challenges in the implementation of an intermediate care model due to relatively unqualified staff. Unlike studies where challenges in collaboration and integration in the whole system have been identified [36–38, 40], this study revealed that there was an especially good mutual understanding of the intermediate role between the intermediate care hospital and the general hospital. Preventing prolonged hospital stays was a shared goal in accordance with national health policy [1]. This is also in line with literature that refers to defining roles and having a shared purpose [55, 56] as essential to achieving successful collaboration. These findings are in contrast to a study describing different commitments, goals and tasks as major obstacles for collaboration between an intermediate unit and the cooperative partners [39].

The reported problem of ‘bedblockers’ [40], which impeded the flow of patients through intermediate care departments, was not found in this study. This was due to the intermediate care hospital's emphasis on being able to release beds for patients ready for discharge from the general hospital and by discharging patients to primary health care on short notice. Although the patients were in a better condition, the short notice discharge resembled the discharge process directly from the general hospital to primary health care, which from the primary health care perspective put the intermediate care hospital in the role of the general hospital [13, 52]. Hence, the intermediate care hospital was perceived more like an extension of hospital than an included part of primary health care.

### Professional communication

Established by earlier studies, communication is regarded as an essential component of teamwork [57] and in interorganisational collaboration [56, 58]. In this study, the general hospital and the intermediate care hospital were satisfied with their way of working together, even if the discharge process presupposed that the intermediate care staff had to seek additional information from the hospital. On the other hand, although sharing information by a common health record, primary health care wished for an improved communication with the intermediate care hospital to obtain a mutual understanding of their role and tasks. Hence, the professionals in the collaborative chain with intermediate care had different views of what is essential information during patients’ discharge. Thus, the intermediate care hospital had not fully solved the communication problem experienced by primary health care without intermediate care. Health professionals still requested a direct communication with the professionals in the organisations discharging patients to them. Similar challenges with respect to differences in professional orientation and context of care have been stated by Paulsen et al. [54]. Information technology might reduce but will not eliminate the need for direct professional communication.

## Conclusions

The professionals' experiences of discharge of elderly in need of continued care via an intermediate care hospital were contrasted favourably compared to discharge directly from the general hospital to primary health care. The intermediate care hospital was perceived as an extension of the hospital and as a buffer for primary health care. The increased capacity to receive and prepare patients was viewed as a benefit. The intermediate care hospital reduced the coordination challenges and eased the pressure on the general hospital as well as the primary health care services. However, inserting intermediate care in the mainstream services did not cover the need for communication between the collaborative partners concerning preparations for further treatment and care in the next location.

Despite a common health record and colocalisation with primary health care services, the intermediate care hospital was experienced more like an extension of hospital than an included part of primary health care. Nevertheless, it is a challenging dual role being an efficient unit that can support early and timely hospital discharge and simultaneously prepare patients in line with primary health care expectations. To fulfil the expectations, an overall process for an integrated care pathway should be established. This implies collaboration with defined roles, tasks and interfaces between the three partners. A well-implemented process may reduce the need for a frequent ad hoc communication between the organisations and thus provide more time for patient care.

Exploring the professionals experiences of intermediate care in the discharge process compared to usual care illustrate opportunities for improvement in similar situations and might be transferrable to other similar settings.

## Authors' contributions

UD conducted the data collection, transcribed the interviews and wrote the drafts of the manuscript. All authors read the focus group interviews, participated in the analysis and critically revised and commended on the drafts of the manuscript. All authors read and approved the final manuscript.

## Figures and Tables

**Box 1.**
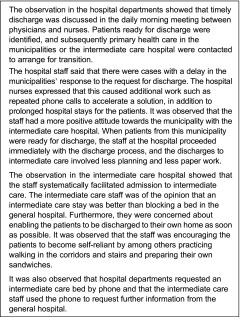
Data from observations

**Table 1.**
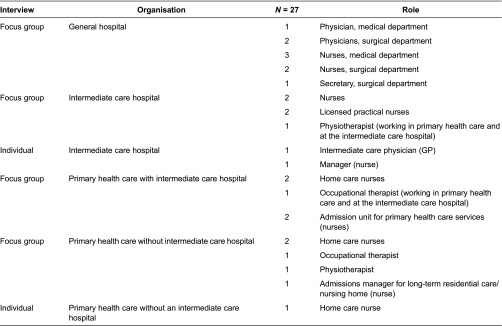
Presentation of participants in interviews by organisation and role
